# Contemporary Fatherhood and Its Consequences for Paternal Psychological Well-being – A Cross-sectional Study of Fathers in Central Europe

**DOI:** 10.3389/fpubh.2016.00199

**Published:** 2016-09-13

**Authors:** Patricia Waldvogel, Ulrike Ehlert

**Affiliations:** ^1^Clinical Psychology and Psychotherapy, University of Zurich, Zurich, Switzerland; ^2^Central European Network on Fatherhood (CENOF), Headquarter at University of Vienna, Vienna, Austria

**Keywords:** family structure, fatherhood, non-residential parenting, parental involvement, psychological well-being

## Abstract

The emotional consequences of fatherhood are markedly conditional on the context in which fatherhood is lived out. This study examines the association between different contemporary forms of fatherhood and paternal psychological well-being. The data are from an anonymous online survey of 3615 biological fathers, stepfathers, adoptive fathers, and foster fathers across the German-speaking countries of Central Europe. First, a detailed characterization of the different existing family constellations is provided. Second, the consequences of these different contemporary forms of fatherhood for paternal psychological well-being are investigated. Fathers of all ages (*M* = 40.11, range: 19–72) with at least one child under the age of 18 were included in the present analysis (*N* = 2785). The presented findings demonstrate that a family structure consisting of two biological parents with biological children seems to be most beneficial to paternal well-being, while some other forms of contemporary fatherhood are associated with impaired well-being, independently of sociodemographic or relationship aspects. More specifically, a history of family separation in non-residential biological fathers and blended-family fathers, and the concomitant loss of father–child contact, is shown to be particularly disadvantageous for the well-being of these fathers. Shared living arrangements, maintaining regular contact with biological children, or forming a new intact family could protect these fathers from negative outcomes.

## Introduction

The healthy development of a child is significantly influenced by paternal care (1). While there has been a great deal of research on the mother–child relationship, the paternal influence on the child, and also the consequences of fatherhood for a man’s life, have received less attention in the literature. Predictors of a fulfilling fatherhood, especially in different contemporary fatherhood contexts, are still relatively unknown. Nevertheless, active involvement of the father in childrearing is nowadays taken for granted in many societies (2, 3), and fathers are much more involved in active childcare than they were some decades ago (4, 5). As father involvement increases, questions about the consequences for a father and his children are gaining in importance.

For the father, the paternal role not only goes along with joy and benefits but can also lead to adverse psychosocial consequences. In general, fatherhood is considered to be both detrimental and rewarding, as having children can enhance social and psychological resources, while at the same time increasing demands and daily strains (6). The transition to fatherhood in particular is a major transformative event, which is associated with a broad range of psychological challenges (7). Besides financial and time costs, the assumption of the father role can be accompanied by an elevated overall stress load (8, 9). In particular, parental role strain or conflict and insecurities regarding the father role may arise pre- and postnatally (10). Moreover, longitudinal assessments have reported a decrease in parental relationship quality and sexual satisfaction and an increased risk of psychopathology (11). However, other studies revealed no changes, or found an increase in mental well-being during and shortly after the transition to fatherhood or following the birth of an additional child (12–14). Cross-sectional studies have also yielded inconsistent results, reporting either positive associations (15–17), negative associations (8, 18, 19), or no associations between fatherhood status and mental well-being (20–23). These inconsistent findings may be explained by the large heterogeneity of the study samples concerning the fathers’ age, marital and socioeconomic status (SES), life stage, age at first birth, co-residence with children, or country of residence (19, 21, 24). Accordingly, the emotional benefits of fatherhood are sometimes outweighed by aspects such as time and financial costs, or a lack of high-quality childcare arrangements (9, 25). Therefore, if fathers are relieved of such structural difficulties, fatherhood becomes more rewarding (25). Additionally, the transformative phase of the transition to fatherhood can be seen as an especially stressful experience, but as family stability increases, other, more positive effects may begin to offset this (2, 10). However, attention should be paid to the fact that all literature cited above rests on evidence from Western societies, such as the U.S., Australia, or Europe. Thus, the experience of fatherhood might be remarkably different in other sociocultural environments (24). In sum, becoming a father is a critical life event, which, under certain contextual prerequisites, provides positive emotional outcomes for fathers in Western societies.

The mental health of the father has a significant influence on his whole family, by affecting his relationship with the mother of his child/children (26), his participation and quality in childrearing (27, 28), and the healthy development of his child or children (27, 29). Taking these aspects into consideration, it is crucial to ascertain under which circumstances fathers are able and willing to invest in their children.

### Pathways for the Emotional Consequences of Fatherhood

As described above, empirical studies from Western societies have yielded inconsistent findings concerning the psychological consequences of having children. Nevertheless, scholarly as well as folk theories predicting emotional advantages of having children are widespread (19). Despite the temporary or situational strains of raising children, having children might represent a fundamental human motive (30), satisfying a human’s basic psychological needs, such as the need for affiliation, positive self-image, or meaning (19), and providing feelings of joy and positive emotion (16, 31). The fulfillment of core psychological needs is crucial for well-being, and its absence can lead to distress and disease (32). Family relationships can also be an important source of social integration and support (10, 15, 25): having children can prevent loneliness and a lack of social support in older age (15, 33, 34) and can buffer the negative effects of the loss of a partner (34). The role accumulation hypothesis suggests that occupying multiple social roles can enhance well-being (34, 35), meaning that the father role can positively influence a man’s well-being irrespective of other occupied roles, such as the work role or the marital role (36). Thus, a positive relationship with one’s children can be an important source of paternal well-being (37, 38), which in the best case scenario, compensates for the negative effects associated with dissatisfaction in other major social roles (23).

Nevertheless, as mentioned above, the context of fatherhood and the concomitant varying role experiences determine whether these beneficial effects ultimately prevail. Role occupancy theory suggests that fatherhood affects men primarily when its roles are clearly occupied (i.e., when dependent children are living in the same household) (39). Accordingly, co-residency with minor children should be especially associated with the profound costs and benefits of fatherhood, while non-custodial or empty-nest fathers should be less affected by their fatherhood status (39). Alternatively, it is also assumed that an active form of fatherhood (e.g., co-residency) is associated with more benefits from the affiliative relationship with the child/children. By contrast, restricted fatherhood, such as in a broken-family situation, may lead to feelings of failure, guilt and role strain, difficulty in fulfilling normative role expectations, and a concomitant loss of the rewarding aspects of the father–child relationship (18, 24, 40). Qualitative studies on divorced fathers’ well-being have already confirmed some of these assumptions (10).

To summarize, fatherhood is seen as a positive role and a developmental milestone in a man’s life. However, some forms of fatherhood are potentially associated with more strains than others. Several psychosocial aspects interrelate with the acquisition of the father role and thus might influence paternal well-being and role satisfaction. Those include sociodemographic characteristics of the father, such as his age, SES, relationship status, family structure, or sociocultural environment; demographic child characteristics, such as child age, gender, or co-residence status; and psychological factors, such as parenting style, social support, or personality traits of father and child (24). Such mechanisms underlying the different associations between fatherhood and well-being, including the father’s family constellation, have to be further investigated (2, 6, 24).

### Paternal Well-being in Contemporary Forms of Fatherhood

Contemporary fatherhood is not limited to the father’s genetic offspring in his current relationship. Due to separation or divorce, some men also take on responsibility for children from previous relationships (e.g., single fathers, non-resident separated fathers), or from multiple partnerships of themselves and/or their partners (blended-family fathers). In step-, foster, or adoptive fatherhood, men also invest in non-genetic offspring. While the literature confirms that children from complex or less “traditional” family models show a higher vulnerability to poor mental and physical health than children from stable, two-biological-parent families (41–43), the consequences of different contemporary family forms for paternal mental health and well-being are less clear. The sparse previous literature on the topic, largely based on data from the U.S., is summarized below. Through a comparison with fathers in traditional two-parent families with biological co-resident children, it focuses on three typical contemporary forms of fatherhood: co-resident single fatherhood, non-resident, living apart fatherhood, and blended- or step-family fatherhood.

Evidence largely suggests that single fathers with children at home have lower psychological well-being than married or cohabiting fathers. Independently of potential SES-related disadvantages in these families, single fathers report more self-perceived stress and depressive symptoms, higher psychiatric morbidity, and lower life satisfaction when compared to stably partnered fathers (8, 9, 17, 18). Thus, while singlehood is generally associated with lower well-being in both men and women compared to being in a steady relationship (44), the lack of a steady partnership seems to be especially burdensome for men with children at home (8).

Fathers who live apart from their minor children, e.g., due to separation or divorce, generally report lower psychological well-being than men living with minor children in the same household (18, 20, 22, 45). The differences are sometimes attributed to partnership status (20, 22), although not in all studies (18, 45). Some researchers concluded that men’s partner history, and not the fathering context, is decisive for their psychological well-being (22). Yet, besides the direct negative effect of relationship dissolution on well-being, separation also has a profound effect on father–child contact and relationship quality in the case of non-residency (46, 47). Additionally, in men living apart from their children, but also in men living with their children, active involvement with the children is associated with higher life satisfaction and fewer depressive symptoms (20, 47). Moreover, longitudinally, the birth of a non-resident child is associated with increased feelings of depression, while the birth of a resident child is not (2). However, factors other than these have been shown to mediate the higher distress in separated, non-resident fathers (46), such as a generally elevated parental role strain or parenting-related stress (48, 49).

Only a small number of studies have examined fathers in step- or blended-family households. In some studies, partnered men living in stepfamilies, with or without additional shared genetic offspring, reported higher levels of depressive symptoms preceding and following the birth of an additional (usually genetic) child when compared to fathers with traditional family models (50). However, after controlling for confounders, no differences were found between fathers with stepchildren at home and men living with biological or adoptive children only, with respect to depression, substance use, and life satisfaction (18, 20, 50, 51).

Taken together, the empirical evidence points to disadvantages concerning the well-being of fathers in some contemporary family forms in Western societies when compared to traditional two-parent families with biological co-resident children. Particularly, non-resident or single fatherhood seem to have detrimental effects for fathers, while blended-family households seem to be less unfavorable. However, apart from sociodemographic and partnership aspects, the reasons for such disadvantages remain relatively unclear. Several of the existing studies on this topic failed to consider important confounding, moderating, or mediating variables, such as a new (non-cohabiting) partnership of the father, multiple-partner fertility, characteristics of the children, or measures of direct childcare activities besides the status of co-residency. Many studies also lacked a clear definition and distinction of the different existing forms of fatherhood, e.g., by only looking at the father’s current household situation, while ignoring his past partnership and fertility history; by mixing up different father types, such as empty nesters and non-custodial fathers living apart from their minor children, or fathers with genetic and non-genetic offspring; or by focusing on one focal child only, instead of considering all of the father’s children from current and past relationships. Therefore, while a significant proportion of children are nowadays raised in multi-core, blended-family forms in Western societies (52, 53), scholars have so far largely neglected to investigate the consequences of such complex family types on fathers’ well-being in detail.

To conclude, even if contextual factors crucially codetermine whether fatherhood is beneficial or costly for a father, there is still a lack of broadly based studies on paternal psychological well-being in the different, complex forms of contemporary fatherhood and across the whole period of fatherhood.

### Aims of the Present Study

The main aim of our study is to ascertain which factors contribute to a satisfying assumption of the father role in the various forms of contemporary fatherhood. In contrast to most existing studies on the topic, we will provide a detailed, distinct description and characterization of the different types of fatherhood, including traditional, separated, single, blended-family, and social fatherhood forms. Controlling for a large number of potential confounding variables, we investigate the consequences of these different contemporary family constellations on multiple facets of paternal psychological well-being. Considering a range of potential moderators and mediators, we wish to determine possible mechanisms underlying the differences in well-being in various father types, including aspects of active paternal involvement and father–child contact. We hypothesize that fathers with stable two-biological-parent families will show higher psychological well-being than fathers with other family forms. We propose that especially fathers with a history of separation from their biological children will show impaired well-being, and that differences between the different father types will be partly mediated by a lower father involvement and father–child contact in some types of fatherhood. Lastly, we hypothesize that forming a new intact family can partly buffer the negative effect of a past history of family separation.

## Materials and Methods

### Participants and Procedure

A total of 4262 males participated in our anonymous online survey on the costs and benefits of fatherhood across the lifespan. A total of 606 respondents were excluded due to missing basic information about their fatherhood status, and a further 41 participants were excluded as they had never fathered any biological or non-biological children, resulting in a total sample of *N* = 3615 fathers. Data were collected in 2014 in the German-speaking part of Central Europe (i.e., Austria, Germany, Switzerland), and the survey was implemented in German language. Participants were recruited *via* announcements in daily newspapers, broadcast and online advertisements, mailing lists of different family- or research-related organizations, and flyers displayed in public places. Inclusion criteria were male sex, a minimum age of 18 years, and having assumed the paternal role for at least one child, i.e., as a biological, step-, foster, or adoptive father, or as the partner of somebody with children. All respondents gave informed consent before completing the online questionnaire. The study was approved by the local Ethics Committee of the Faculty of Arts, University of Zurich, Switzerland before recruitment started.

For the present analysis, fathers were included if they had listed at least one child under the age of 18 years. A total of 3031 fathers met this criterion, of which 2536 (83.7%) fathers filled in the complete questionnaire, while the remaining 495 (16.3%) only provided incomplete data. Of those who provided incomplete data, 246 participants could not be assigned to one of the father types applied in the present analysis due to missing or invalid data on at least one relevant question and therefore had to be excluded from the analysis. In the remaining sample, study dropout before completing the whole questionnaire was not related to certain types of fatherhood [χ^2^(4) = 4.46, *p* = 0.347]. Most participants were from Switzerland (60.7%), followed by Germany (22.2%), Austria (15.9%), and other neighboring countries (1.2%), e.g., Liechtenstein or Italy. The mean age of fathers was 40.11 years (SD = 7.68, range: 19–72), and the age of their children ranged from 0 to 52 years. Most fathers were married (65.4%) or in a common-law relationship (18.6%). The majority listed biological children only (85.7%), while the remaining fathers reported either biological and non-biological children (12.1%) or non-biological children only (2.1%). With 49.9% of participants having completed tertiary or higher education, our sample is moderately selective in terms of a higher SES compared to the general population of males (54).

### Dependent Variables: Psychological Well-being

Psychological well-being was measured on three dimensions, representing different positive (life satisfaction) and negative (chronic stress, psychological distress) facets of well-being.

#### Chronic Stress

Perceived chronic stress was assessed using the Screening Scale of Chronic Stress (SSCS) of the Trier Inventory for the Assessment of Chronic Stress (TICS) (55). The SSCS is a 12-item self-rating scale assessing the frequency of experiencing chronic worry, work and social overload, excessive demands, and lack of social recognition during the past 3 months. Responses to each item were given on a 5-point rating scale, ranging from 0 (never) to 4 (very often). The SSCS sum score represents an overall value for global, unspecific stress load, ranging from 0 (no stress) to 48 (very frequent stress). The SSCS shows good internal consistency (Cronbach’s α = 0.91) and satisfactory convergent and discriminant validity (55).

#### Psychological Distress

Psychological distress was measured using the German translation of the short version of the Brief Symptom Inventory (BSI-18) (56). This internationally used, reliable, and valid self-report measure assesses symptom severity on the dimensions of depression, somatization, and anxiety with six items each, during the past 7 days. Each item was scored on a 5-point Likert scale from 0 (not at all) to 4 (extremely). The sum scores of the three subscales can be aggregated to a Global Severity Index (GSI), representing a measure of general psychological distress ranging from 0 to 72.

#### Life Satisfaction

Subjective life satisfaction was assessed by a single question asking respondents how satisfied they are with their lives in general. Answers were given on a visual analog scale (VAS) ranging from 0 (not satisfied at all) to 100 (very satisfied). Research has shown that single-item measures of life satisfaction show a satisfactory level of reliability (57).

### Independent Variables: Family Constellation

#### Partnership and Fertility History

To determine the partnership history, participants were asked questions about their marital status (“unmarried,” “divorced/separated,” “widowed,” “married”) and their current relationship status (“no committed relationship,” “committed, non-cohabiting relationship,” “committed, cohabiting relationship”). For fatherhood status and fertility history, participants reported how many biological children they have, for how many non-biological children (i.e., stepchildren/children of partner, adoptive, or foster children) they have assumed the paternal role, and from how many different partners the reported children were. For the present analyses, step-, adoptive, and foster children were all subsumed under the term “non-biological children.” Contribution to caring for close relatives, such as younger siblings or grandchildren, without legally being entitled to custody, was not defined as being in a father role in the present study.

#### Characteristics of the Children

For each listed child, fathers provided individual information about the child’s age and gender, their relationship to the child (“biological child,” “stepchild/child of (ex-)partner,” “adoptive child,” “foster child,” “other”) and to his or her mother (“current partner/wife,” “ex-partner/ex-wife,” “sexual relationship,” “other”), and the co-residence status with the child (“living together full-time,” “living together part-time,” “not living together”).

#### Paternal Involvement

Beyond co-residence status, to measure paternal involvement, fathers were asked to report their average amount of contact with each child during the past 12 months (“no contact,” “sporadic contact,” “once a month,” “every two weeks,” “weekly,” “more than weekly,” “daily”). An average contact of less than once a month was defined as a lack of regular contact. Additionally, fathers stated how many hours a week they usually spent on active childcare activities.

#### Different Forms of Contemporary Fatherhood

**Figure 1:**
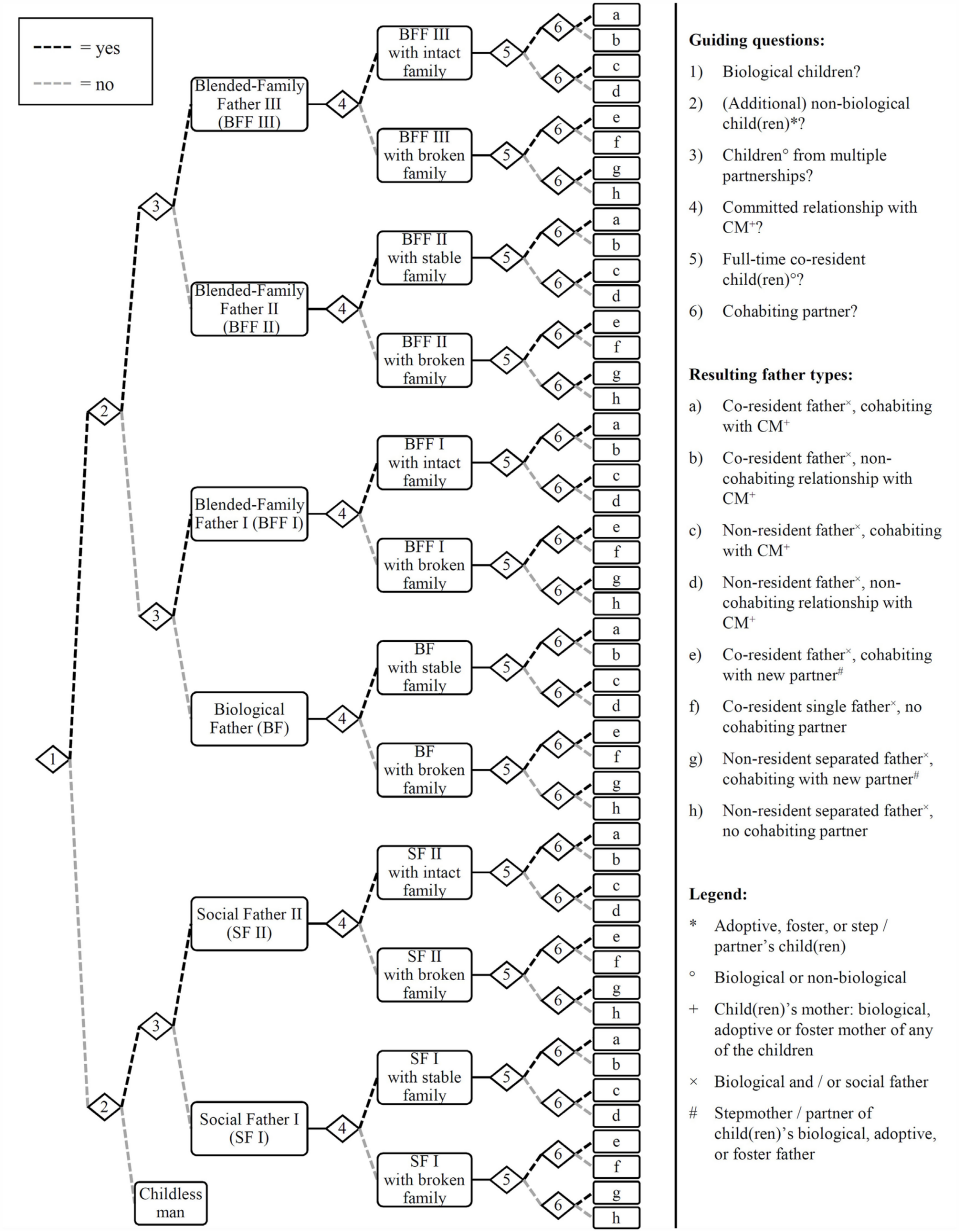
**Contemporary forms of fatherhood**.

Based on their individual description about their fatherhood status and fertility history, their relationship to each child and his or her mother, and their co-residence status with the current partner and children, participants were categorized into different father types (Figure 1). Five main subgroups were identified for data analysis: (1) Biological (bio) fathers with stable families (BF: a–d) (only biological children with current partner/wife); (2) Separated bio fathers (BF: g–h) (only non-resident or part-time co-resident biological children from previous partnership); (3) Blended-family fathers (BFF: a–d, g–h) (biological and non-biological children, or biological children from multiple partnerships; from current and/or past partnership/s); (4) Single fathers (BF/BFF/SF: f) (full-time co-resident children; no cohabiting partner); and (5) Social fathers (SF: a–d, g–h) (non-biological children only; from current and/or past partnership/s). Co-resident fathers cohabiting with a new partner without her own children (BF/BFF/SF: e) are not included in the present analysis due to a small sample size.

### Other Influencing Variables

#### Sociodemographic Factors

All participants provided information about potentially confounding sociodemographic influences, such as age (years), education (0 = “non-tertiary,” 1 = “tertiary”), personal and household income (CHF/month), occupation (0 = “paid work” or “stay-at-home,” 1 = “unoccupied”), and workload (0 = “≤30 h/week,” 1 = “>30 h/week”).

#### Relationship Satisfaction

In participants who stated being in a committed relationship, relationship satisfaction was assessed using the German version of the Relationship Assessment Scale (RAS) (58). The 7 items describe different aspects of relationship satisfaction on a 5-point Likert scale. The mean value across all items represents a measure of generic relationship satisfaction from 1 (not satisfied at all) to 5 (very satisfied).

### Statistical Analysis

All statistical analyses were conducted using SPSS, version 22.0 (IBM, Armonk, NY, USA). To test for the effect of father type on well-being, multiple ordinary least squares (OLS) regression was performed for each outcome: Model 1 includes the dummy variables representing different father types while controlling for sociodemographic factors of fathers and children; relationship aspects were entered into Model 2a/b; characteristics of active fathering were entered into Model 3a/b. For moderating effects, additional interaction terms were added to the models where appropriate. Simple mediation analyses for group differences were conducted using OLS path analysis with PROCESS for SPSS, version 2.15 (59). After Model 2, only father types that still differed from the reference category after controlling for confounders were considered for further analyses. Hypothesis testing is based on bootstrap SEs and confidence intervals based on 1000 samples in all analyses due to distribution problems in some variables. All statistical tests were two-tailed. Statistical significance was evaluated at *p* ≤ 0.05, with *p* ≤ 0.10 being considered as borderline significant.

## Results

### Descriptive Statistics

The distribution of participants across the different forms of contemporary fatherhood is presented in Table 1. The means and SDs of all variables included in the analysis, or absolute and percentage frequencies for dichotomous variables, respectively, divided by different father types, are presented in Table 2.

**Table 1:** **Distribution of participants across different forms of contemporary fatherhood**.

	BF	BFF I	BFF II	BFF III	SF I	SF II
(a) Co-resident father, cohabiting with CM	*N* = 1890	*N* = 105	*N* = 77	*N* = 113	*N* = 36	*N* = 3
	67.9%	3.8%	2.8%	4.1%	1.3%	0.1%
(b) Co-resident father, non-cohabiting relationship with CM	*N* = 5	*N* = 2	–	*N* = 4	–	–
	0.2%	0.1%		0.1%		
(c) Non-resident father, cohabiting with CM	*N* = 8	*N* = 2	*N* = 2	*N* = 9	*N* = 3	–
	0.3%	0.1%	0.1%	0.3%	0.1%	
(d) Non-resident father, non-cohabiting relationship with CM	*N* = 10	–	–	*N* = 19	*N* = 4	–
	0.4%			0.7%	0.1%	
(e) Co-resident father, cohabiting with new partner	*N* = 15	–	*N* = 2	*N* = 3	–	–
	0.5%		0.1%	0.1%		
(f) Co-resident single father, no cohabiting partner	*N* = 53	*N* = 7	*N* = 6	*N* = 15	–	–
	1.9%	0.3%	0.2%	0.5%		
(g) Non-resident separated father, cohabiting with new partner	*N* = 72	*N* = 4	*N* = 9	*N* = 11	*N* = 2	*N* = 1
	2.6%	0.1%	0.3%	0.4%	0.1%	0.0%
(h) Non-resident separated father, no cohabiting partner	*N* = 196	*N* = 19	*N* = 26	*N* = 42	*N* = 7	*N* = 3
	7.0%	0.7%	0.9%	1.5%	0.3%	0.1%

*BF, bio father; BFF, blended-family father; SF, social father; CM, children’s mother*.

**Table 2:** **Means and SDs or absolute and percentage frequencies in total sample and by different father types**.

Variables	Total (*N* = 2785)	Bio fathers with stable families (*N* = 1913)	Separated bio fathers (*N* = 268)	Blended-family fathers (*N* = 444)	Single fathers (*N* = 81)	Social fathers (*N* = 59)
**Sociodemographic characteristics of father and children**						
Age (19–72)	40.11 (7.68)	38.95 (6.99)	41.94 (7.62)	43.32 (8.62)	44.53 (7.53)	39.47 (10.39)
Education *N* (%)						
Primary/secondary^a^	1394 (49.9)	854 (44.6)	159 (59.3)	287 (64.6)	52 (64.2)	31 (52.5)
Tertiary^a^	1391 (50.1)	1059 (55.4)	109 (40.7)	157 (35.4)	29 (35.8)	28 (47.5)
Household income (121–36,000)	6944 (4598)	7617 (4481)	5590 (4868)	5602 (4361)	4236 (3804)	5384 (4121)
Occupation *N* (%)						
Paid work/stay-at-home^b^	2734 (98.2)	1901 (99.4)	258 (96.3)	426 (95.9)	73 (90.1)	56 (94.9)
Unoccupied^b^	51 (1.8)	12 (0.6)	10 (3.7)	18 (4.1)	8 (9.9)	3 (5.1)
Workload *N* (%)						
≤30 h/week^c^	626 (22.5)	379 (19.8)	66 (24.6)	110 (24.8)	40 (49.4)	21 (35.6)
>30 h/week^c^	2159 (77.5)	1534 (80.2)	202 (75.4)	334 (75.2)	41 (50.6)	38 (64.4)
Number of children (1–12)	1.98 (1.07)	1.74 (0.79)	1.56 (0.75)	3.22 (1.23)	2.56 (1.64)	1.69 (0.84)
Age of youngest child (0–17)	4.88 (4.55)	3.95 (4.14)	8.12 (4.34)	5.63 (4.53)	9.30 (4.99)	7.56 (4.34)
**Partnership aspects**						
Relationship status *N* (%)						
No committed relationship^d^	320 (11.5)	19 (1.0)	146 (54.5)	79 (17.8)	55 (67.9)	12 (20.3)
Committed, non-cohabiting^d^	124 (4.5)	6 (0.3)	53 (19.8)	35 (7.9)	26 (32.1)	4 (6.8)
Committed, cohabiting^d^	519 (18.6)	307 (16.0)	57 (21.3)	124 (27.9)	0 (0.0)	21 (35.6)
Committed, marital^d^	1822 (65.4)	1581 (82.6)	12 (4.5)	206 (46.4)	0 (0.0)	22 (37.3)
Relationship satisfaction (1.1–5.0)	4.13 (0.68)	4.14 (0.66)	4.06 (0.77)	4.10 (0.70)	3.99 (0.77)	4.02 (0.65)
**Paternal involvement**						
Active childcare/week (0–168)	36.78 (24.99)	37.09 (22.84)	31.17 (24.57)	36.07 (27.85)	60.14 (42.04)	24.59 (21.26)
Co-resident bio children *N* (%)						
None^e^	283 (10.2)	5 (0.3)	119 (44.4)	100 (22.5)	0 (0.0)	59 (100.0)
Part-time^e^	233 (8.4)	13 (0.7)	149 (55.6)	71 (16.0)	0 (0.0)	–
Full-time^e^	2269 (81.5)	1895 (99.1)	0 (0.0)	273 (61.5)	81 (100.0)	–
Co-resident non-bio children *N* (%)						
None^f^	2592 (93.1)	1913 (100.0)	268 (100.0)	299 (67.3)	79 (97.5)	15 (25.4)
Part-time^f^	29 (1.0)	–	–	23 (5.2)	0 (0.0)	5 (8.5)
Full-time^f^	164 (5.9)	–	–	122 (27.5)	2 (2.5)	39 (66.1)
Number of bio children without regular contact (0–4)	0.07 (0.35)	0.00 (0.07)	0.09 (0.34)	0.34 (0.74)	0.20 (0.49)	–
Number of non-bio children without regular contact (0–6)	0.09 (0.42)	–	–	0.46 (0.83)	0.32 (0.95)	0.25 (0.54)
**Paternal well-being**						
Chronic stress (0–48)	14.70 (8.38)	13.94 (7.95)	17.13 (9.19)	15.78 (8.78)	17.09 (10.08)	15.68 (8.50)
Psychological distress (0–56)	5.99 (6.75)	5.11 (5.43)	9.00 (9.11)	7.38 (8.49)	8.11 (8.05)	5.91 (6.78)
Life satisfaction (4–100)	76.22 (17.24)	79.35 (14.31)	66.60 (21.31)	71.52 (19.71)	64.21 (23.27)	73.58 (19.75)

*Variable ranges are noted in parentheses following the names of the variables. Table entries are means and, in parentheses, SDs, or absolute and percentage frequencies where noted*.

*^a–f^Variables are mutually exclusive*.

### Well-being in Different Father Types

First, the direct association between father type and well-being was tested. As hypothesized, without controlling for confounders, bio fathers with stable families reported significantly higher psychological well-being on all three measured dimensions (i.e., chronic stress, life satisfaction, and psychological distress) when compared to separated bio fathers, blended-family fathers, and single fathers (Table 2; all *p* ≤ 0.01). Social fathers did not vary significantly from bio fathers with stable families regarding chronic stress and psychological distress but reported lower life satisfaction (*p* ≤ 0.05).

However, we wished to investigate which of these group differences remain stable independently of differences in sociodemographic characteristics or partnership aspects. Table 3 shows the results of the OLS regressions in which fathers’ well-being was regressed on dummy-coded father types, while controlling for individual, sociodemographic characteristics of the fathers and their children in a first step (Model 1), and partnership aspects in a second step (Model 2a/b). Bio fathers with stable families are the reference category for all models.

**Table 3:** **Well-being in different father types, controlled for sociodemographic and partnership aspects**.

Variables	Chronic stress (*N* = 2596)			Life satisfaction (*N* = 2739)			Psychological distress (*N* = 2612)		
	Model 1	Model 2a	Model 2b	Model 1	Model 2a	Model 2b	Model 1	Model 2a	Model 2b
**Father type**									
Bio fathers with stable families^a^	Ref.	Ref.	Ref.	Ref.	Ref.	Ref.	Ref.	Ref.	Ref.
Separated bio fathers^a^	2.53*** (1.21, 3.83)	0.27 (−1.38, 2.13)	1.87^†^ (−0.06, 3.85)	−10.61*** (−13.52, −7.85)	−1.73 (−4.94, 1.55)	−4.79** (−8.30, −1.51)	3.32*** (2.14, 4.56)	0.97 (−0.78, 2.69)	2.23* (0.25, 4.38)
Blended-family fathers^a^	0.95^†^ (−0.08, 2.00)	0.10 (−0.98, 1.22)	0.50 (−0.51, 1.60)	−6.95*** (−9.02, −4.75)	−3.87*** (−5.98, −1.66)	−4.32*** (−6.33, −2.43)	1.82*** (0.97, 2.72)	1.14* (0.18, 2.09)	1.40*** (0.52, 2.26)
Single fathers^a^	1.55 (−0.83, 3.77)	−1.29 (−3.93, 1.30)	−2.31 (−5.61, 0.97)	−11.18*** (−16.19, −6.10)	−0.29 (−5.87, 5.09)	1.16 (−6.13, 7.65)	1.47^†^ (−0.15, 3.18)	−1.54 (−3.80, 0.54)	−1.06 (−3.87, 1.58)
Social fathers^a^	1.10 (−1.39, 3.74)	0.25 (−2.21, 2.95)	0.51 (−2.11, 3.47)	−3.64 (−8.80, 0.91)	−0.17 (−5.16, 4.53)	−2.49 (−6.87, 1.53)	0.31 (−1.53, 2.42)	−0.42 (−2.23, 1.52)	−0.09 (−1.92, 2.05)
**Partnership status**									
No committed relationship^a^		Ref.	–		Ref.	–		Ref.	–
Non-cohabiting relationship^a^		−0.27 (−2.19, 1.75)	Ref.		9.45*** (5.02, 13.44)	Ref.		−1.55 (−3.61, 0.46)	Ref.
Cohabiting relationship^a^		−2.33** (−3.82, −0.85)	−1.35 (−3.45, 0.71)		12.52*** (9.12, 15.80)	3.51* (0.27, 6.75)		−3.72*** (−5.40, −2.07)	−1.93^†^ (−4.01, 0.25)
Marital relationship^a^		−3.16*** (−4.70, −1.63)	−1.26 (−3.29, 0.85)		14.78*** (11.30, 18.16)	3.41* (0.22, 6.83)		−3.70*** (−5.46, −2.03)	−1.25 (−3.30, 0.96)

Relationship satisfaction			−3.38*** (−3.93, −2.88)			10.51*** (9.67, 11.47)			−2.54*** (−2.97, −2.08)

Total adjusted *R*^2^	0.036	0.043	0.106	0.122	0.158	0.272	0.059	0.074	0.113

*OLS regressions. Numbers represent unstandardized coefficients, with 95% confidence intervals listed in parentheses. Results are based on 1000 bootstrap samples. All models control for fathers’ age, education, household income, occupation, workload, number of children, and age of youngest child*.

*Ref. = reference category*.

*^a^0 = no, 1 = yes*.

*^†^p ≤ 0.10*.

*p ≤ 0.05.

**p ≤ 0.01.

***p ≤ 0.001 (two-tailed tests).

After controlling for individual sociodemographic characteristics of the fathers and their children (Model 1), separated bio fathers and blended-family fathers were still more likely to report lower psychological well-being than bio fathers with stable families on all three dimensions. They reported significantly higher chronic stress (blended-family fathers: borderline significant) and psychological distress and significantly lower life satisfaction when compared to bio fathers with stable families. Single fathers also still reported significantly lower life satisfaction and a tendency toward higher psychological distress, while differences in life satisfaction between bio fathers with stable families and social fathers vanished after controlling for individual characteristics.

We then examined whether the remaining group differences could be attributed to differences in partnership aspects between groups by controlling for relationship and marital status (Model 2a) and relationship satisfaction (Model 2b). After controlling for relationship and marital status, only blended-family fathers still showed significantly lower psychological well-being (psychological distress and life satisfaction) in comparison to the reference category, while no difference was found for separated bio fathers and single fathers. However, after adding relationship satisfaction to the model for those fathers who were in a relationship (Model 2b), separated bio fathers and blended-family fathers both varied significantly from the reference category regarding psychological well-being [separated bio fathers: chronic stress (borderline significant), psychological distress, and life satisfaction; blended-family fathers: psychological distress and life satisfaction].

### Active Fathering and Well-being in Separated Bio Fathers and Blended-Family Fathers

We then wished to determine whether aspects of active fathering predict well-being in separated bio fathers and blended-family fathers, and if so, whether these aspects account for group differences in well-being between these fathers and bio fathers with stable families. To this aim, we first combined separated bio fathers and blended-family fathers into one group. The same predictors as before were entered into our models in a first step, and different aspects of active fathering were added in a second step (Model 3a). Interaction terms between contact and co-residency with children were added in an additional step (Model 3b) to clarify whether co-residency with some children can buffer negative effects of a lack of contact with other children.

As shown in Table 4, the total amount of childcare activities per week was not directly associated with psychological well-being in these fathers. However, the number of biological children without regular contact was significantly negatively associated with paternal well-being on all three dimensions. Fathers maintaining regular contact with all of their biological children reported significantly higher life satisfaction, a significantly lower chronic stress load, and significantly less psychological distress than fathers with a loss of regular contact with some of their children. Furthermore, part-time or full-time co-residency with biological children independently predicted psychological well-being: fathers with biological children living in their household showed significantly higher life satisfaction (part-time and full-time), less psychological distress [part-time (borderline significant) and full-time], and less chronic stress (full-time) in comparison to fathers with no co-resident biological children. Regarding paternal involvement with non-biological children, part-time co-residency with non-biological children was significantly negatively related to paternal psychological distress. Contact or full-time co-residency with non-biological children did not seem to be related to any aspect of paternal well-being. Contact with biological children significantly interacted with part-time or full-time co-residency with biological children: the negative effect of a lack of contact with some biological children on life satisfaction and psychological distress was less distinct in fathers who reported co-residing with other biological children than in those without any co-resident biological children (life satisfaction: part-time and full-time; psychological distress: full-time). Co-residency with non-biological children also significantly interacted with contact with biological children, but in a negative manner: the negative effect of a lack of contact with biological children on life satisfaction was more distinct in fathers with non-biological children living full-time in their household than in those without any co-resident non-biological children. Adding relationship satisfaction as a covariate to all models did not fundamentally change the findings. Therefore, models without the inclusion of relationship satisfaction are presented here.

**Table 4:** **Paternal involvement and well-being in separated bio fathers and blended-family fathers**.

Variables	Chronic stress (*N* = 663)		Life satisfaction (*N* = 689)		Psychological distress (*N* = 665)	
	Model 3a	Model 3b	Model 3a	Model 3b	Model 3a	Model 3b
Active childcare/week	−0.02 (−0.04, 0.01)	−0.02 (−0.04, 0.01)	0.01 (−0.06, 0.08)	0.01 (−0.06, 0.08)	0.00 (−0.03, 0.03)	0.01 (−0.02, 0.03)
**Number of children without regular contact**						
Biological	1.12* (0.14, 2.12)	1.97* (−0.03, 3.72)	−4.41** (−6.90, −2.12)	−7.36** (−12.20, −1.72)	1.30* (0.19, 2.51)	2.90** (0.27, 4.85)
Non-biological	−0.40 (−1.92, 0.96)	−0.43 (−1.91, 0.96)	−1.42 (−4.20, 1.42)	−1.34 (−4.23, 1.40)	0.24 (−1.44, 1.63)	0.27 (−1.37, 1.61)
**Co-residency with children**						
Biological, none^a^	Ref.	Ref.	Ref.	Ref.	Ref.	Ref.
Biological, part-time^a^	−1.45 (−3.35, 0.44)	−1.02 (−3.03, 0.87)	5.27* (1.38, 9.27)	3.79^†^ (−0.43, 8.16)	−1.85^†^ (−3.87, 0.11)	−1.50 (−3.59, 0.47)
Biological, full-time^a^	−3.60** (−6.03, −1.09)	−2.95* (−5.61, −0.21)	6.59** (1.18, 11.15)	4.01 (−0.98, 8.85)	−3.83** (−6.28, −1.50)	−2.66* (−5.35, −0.40)
Non-biological, none^a^	Ref.	Ref.	Ref.	Ref.	Ref.	Ref.
Non-biological, part-time^a^	−2.03 (−6.34, 2.65)	−2.60 (−7.01, 1.90)	−2.27 (−10.66, 5.32)	−1.28 (−10.26, 6.68)	−3.63* (−6.75, −0.27)	−4.03* (−7.17, −0.65)
Non-biological, full-time^a^	−1.22 (−3.97, 1.63)	−1.54 (−4.24, 1.33)	1.61 (−3.89, 7.21)	3.40 (−2.14, 9.16)	−1.41 (−3.80, 1.01)	−1.70 (−4.00, 0.84)
**Co-residency with children × number of biological children without regular contact (= “contact”)**						
Bio part-time × contact		−2.53 (−5.53, 1.21)		8.60** (1.54, 15.03)		−1.43 (−4.90, 3.40)
Bio full-time × contact		−1.47 (−3.44, 0.80)		5.99* (−0.02, 11.05)		−3.03** (−4.90, −0.27)
Non-bio part-time × contact		2.76 (−15.45, 8.97)		−4.47 (−13.18, 3.41)		1.00 (−7.02, 5.58)
Non-bio full-time × contact		0.97 (−1.27, 3.25)		−6.39* (−13.85, −1.16)		0.88 (−1.47, 3.33)

*R*^2^ change for new predictors	0.028**	0.005	0.036***	0.016**	0.034***	0.010

Total adjusted *R*^2^	0.082	0.082	0.196	0.207	0.097	0.102

*OLS regressions. Numbers represent unstandardized coefficients, with 95% confidence intervals listed in parentheses. Results are based on 1000 bootstrap samples. All models control for fathers’ age, education, household income, occupation, workload, relationship status, number of children, age of youngest child, and non-biological children = yes/no. Ref. = reference category*.

*^a^0 = no, 1 = yes*.

*^†^p ≤ 0.10*.

*p ≤ 0.05.

**p ≤ 0.01.

***p ≤ 0.001 (two-tailed tests).

For mediation analyses, we compared the group of bio fathers with stable families with the groups of separated bio fathers and blended-family fathers, respectively, to estimate whether group differences can be explained by differences in contact with biological children. As the lack of contact with biological children was found to most profoundly affect well-being in fathers without co-resident biological children in their household (Table 4), we divided separated bio fathers and blended-family fathers into subgroups with or without biological children living at least part-time in their household. We then compared these subgroups to bio fathers with stable families, while controlling for potential confounders in all path models.

When comparing separated bio fathers to bio fathers with stable families, differences in well-being between father types remained completely unexplained by the lack of contact with children, regardless of whether or not separated fathers had any part-time co-resident children in their household (Table 5a,b). Separated bio fathers without part-time co-resident children were significantly more likely to experience a lack of contact with their children, chronic stress, psychological distress (borderline significant), and lower life satisfaction compared to bio fathers with stable families (Table 5a). However, these differences in the amount of contact with children did not significantly mediate the differences in well-being between father types. Bootstrap confidence intervals for the indirect effects all contained zero, indicating no significant indirect effect of father type on well-being *via* the amount of regular contact with biological children. For separated bio fathers with part-time co-resident children, contact with biological children and psychological well-being did not differ significantly from bio fathers with stable families (Table 5b).

**Table 5:** **Simple mediation analysis for well-being in bio fathers with stable families versus separated bio and blended-family fathers, mediated by lack of contact with biological children**.

Outcome:	M (Contact^b^)	Y (Chronic stress)	M (Contact^b^)	Y (Life satisfaction)	M (Contact^b^)	Y (Psychol. distress)
Independent variables:						
**Model (a) Separated bio fathers without co-resident children**						
X (Father type^a^)	*a* = 0.15** (0.04, 0.26)	*c*′ = 4.48** (1.19, 7.76)	*a* = 0.14** (0.04, 0.25)	*c*′ = −8.19** (−13.24, −3.14)	*a* = 0.13* (0.03, 0.23)	*c*′ = 3.12^†^ (−0.05, 6.29)
M (Contact^b^)	–	*b* = 1.85 (−3.98, 7.68)	–	*b* = 1.71 (−3.75, 7.16)	–	*b* = 0.88 (−4.78, 6.54)
Total *R*^2^	0.088	0.120***	0.079	0.304***	0.071	0.123***
	*N* = 1852		*N* = 1947		*N* = 1862	
**Model (b) Separated bio fathers with part-time co-resident children**						
X (Father type^a^)	*a* = 0.02 (−0.03, 0.07)	*c*′ = 1.48 (−1.18, 4.14)	*a* = 0.02 (−0.03, 0.07)	*c*′ = −2.57 (−7.22, 2.08)	*a* = 0.02 (−0.03, 0.07)	*c*′ = 2.27 (−0.45, 4.99)
M (Contact^b^)	–	*b* = −0.55 (−4.95, 3.86)	–	*b* = 4.89 (−5.15, 14.94)	–	*b* = 0.33 (−4.46, 5.12)
Total *R*^2^	0.032	0.114***	0.029	0.293***	0.030	0.110***
	*N* = 1877		*N* = 1971		*N* = 1887	
**Model (c) Blended-family fathers without co-resident children**						
X (Father type^a^)	*a* = 0.52*** (0.27, 0.78)	*c*′ = 1.14 (−1.56, 3.85)	*a* = 0.52*** (0.28, 0.75)	*c*′ = −8.12** (−13.99, −2.24)	*a* = 0.52*** (0.27, 0.77)	*c*′ = 1.66 (−1.07, 4.40)
M (Contact^b^)	–	*b* = 3.91*** (1.59, 6.22)	–	*b* = −8.10** (−13.62, −2.59)	–	*b* = 4.17*** (1.83, 6.51)
Total *R*^2^	0.262**	0.132***	0.286**	0.326***	0.258*	0.153***
	*N* = 1851		*N* = 1948		*N* = 1863	
**Model (d) Blended-family fathers with part-time or full-time co-resident children**						
X (Father type^a^)	*a* = 0.19*** (0.13, 0.26)	*c*′ = −0.05 (−1.22, 1.13)	*a* = 0.18*** (0.12, 0.23)	*c*′ = −3.85*** (−5.98, −1.73)	*a* = 0.19*** (0.13, 0.26)	*c*′ = 1.07* (0.10, 2.04)
M (Contact^b^)	–	*b* = −0.08 (−1.23, 1.06)	–	*b* = −0.43 (−2.44, 1.57)	–	*b* = −0.35 (−1.32, 0.63)
Total *R*^2^	0.170***	0.114***	0.158***	0.290***	0.169***	0.122***
	*N* = 2087		*N* = 2201		*N* = 2105	

*OLS path analyses. Numbers represent unstandardized coefficients, with 95% confidence intervals listed in parentheses. Results are based on 1000 bootstrap samples. All models control for fathers’ age, education, household income, occupation, workload, relationship status and satisfaction, number of children, and age of youngest child. ab = indirect effects, c′ = direct effects*.

*^a^0 = bio fathers with stable families, 1 = separated bio fathers (Model a, b) or blended-family fathers (Model c, d)*.

*^b^Number of biological children without regular contact*.

*^†^p ≤ 0.10*.

*p ≤ 0.05.

**p ≤ 0.01.

***p ≤ 0.001 (two-tailed tests).

For blended-family fathers compared to bio fathers with stable families, the number of biological children with a lack of regular contact significantly mediated differences in well-being between father types. This mediation effect was conditional on co-residence status, being only significant in those blended-family fathers without any co-resident biological children. As can be seen in Table 5c, these fathers reported a significantly higher number of biological children without regular contact, and a lack of regular contact significantly predicted lower paternal life satisfaction, a higher chronic stress load, and more psychological distress. Bias-corrected bootstrap confidence intervals for the indirect effects (*ab* = −4.20/2.04/2.17) based on 1000 bootstrap samples did not contain zero (−8.228 to −1.227/0.711 to 4.077/0.530 to 4.055), indicating a statistically significant indirect effect of father type on well-being *via* the amount of regular contact with biological children. Additionally, there was evidence that father type significantly influenced well-being *via* other pathways, which are independent of its effect on the lack of contact with biological children [Table 5c: *c*′ (life satisfaction)]. Blended-family fathers with (part-time) co-resident biological children also reported significantly decreased contact with some of their biological children, significantly lower life satisfaction, and significantly more psychological distress compared to bio fathers with stable families (Table 5d). However, the lack of contact with biological children did not mediate impaired well-being in these fathers, as indicated by bootstrap confidence intervals, which all contained zero for the indirect effects.

### Buffering Effect of Forming a New Intact Family after Family Separation

Lastly, we investigated whether forming a new intact family (i.e., a committed relationship with shared biological or non-biological children) can partly buffer the negative effect of a past history of family separation in blended-family fathers, despite the potential loss of contact with children from past relationships. Therefore, blended-family fathers with intact families and a history of family separation (Figure 1, BFF I/III: a–d, BFF I/II/III: g–h) were compared to blended-family fathers living in broken-family contexts, blended-family fathers with stable families (i.e., without a history of separation: Figure 1, BFF II: a–d), separated bio fathers, and bio fathers with stable families. As can be seen in Table 6, controlling for sociodemographic and relationship aspects, blended-family fathers with intact families reported a significantly lower level of chronic stress and psychological distress than blended-family fathers with broken families, or separated bio fathers (borderline significant). At the same time, they did not differ significantly from bio fathers or blended-family fathers with stable families on these dimensions of well-being. By contrast, blended-family fathers with intact families did not reach the level of life satisfaction reported by bio fathers or blended-family fathers with stable families, as indicated by significant differences in life satisfaction when compared to these subgroups, while reporting comparable degrees of life satisfaction as blended-family fathers or bio fathers with broken families.

**Table 6:** **Well-being in blended-family fathers with a history of family separation after forming a new intact family**.

Variables	Chronic stress *N* = 2246	Life satisfaction *N* = 2364	Psychological distress *N* = 2259
Blended-family fathers with intact families^a^	Ref.	Ref.	Ref.
Bio fathers with stable families^a^	−0.30 (−1.72, 1.04)	5.03*** (2.31, 7.89)	−0.77 (−2.03, 0.31)
Separated bio fathers^a^	2.17^†^ (−0.17, 4.50)	−1.16 (−5.39, 2.89)	2.11^†^ (−0.04, 4.29)
Blended-family fathers with stable families^a^	−0.00 (−2.14, 2.05)	3.89* (0.25, 7.47)	0.21 (−1.34, 1.91)
Blended-family fathers with broken families^a^	3.40* (0.07, 6.48)	−4.84 (−10.87, 1.75)	4.49* (1.24, 7.93)

*OLS regressions. Numbers represent unstandardized coefficients, with 95% confidence intervals listed in parentheses. Results are based on 1000 bootstrap samples. All models control for fathers’ age, education, household income, occupation, workload, relationship status and satisfaction, number of children, and age of youngest child. Ref. = reference category*.

*^a^0 = no, 1 = yes*.

*^†^p ≤ 0.10*.

*p ≤ 0.05.

***p ≤ 0.001 (two-tailed tests).

## Discussion

The aim of our study was to provide a detailed characterization of the different existing family constellations and to ascertain which factors contribute to a satisfying assumption of the father role in the various forms of contemporary fatherhood observed in Central European countries. Consistent with the literature, our main findings indicate that biological fathers with stable families show the highest well-being when compared to other, less traditional family forms. As hypothesized, while sociodemographic and partnership aspects are substantial predictors of well-being in all types of fathers, in particular, those with a history of separation from some or all of their biological children (i.e., separated bio fathers and most types of blended-family fathers) independently seem to have impaired psychological well-being on multiple dimensions. This is in line with previous cross-sectional and longitudinal studies showing that fathers living apart from their children due to family separation show a worse outcome independently of sociodemographic or relationship aspects (18, 45). Moreover, we found that impaired well-being in social or single fathers could be attributed to individual sociodemographic characteristics or relationship status of these fathers. Thus, as described in other cross-sectional, but also longitudinal studies, living in families with non-biological children, with or without additional biological children, does not seem to be particularly burdensome, aside from a generally lower SES in these types of family constellations (18, 20, 50), while living without a partner seems to be specifically burdensome in custodial fatherhood (8).

Regarding reasons for impaired well-being in separated or blended-family fathers, our findings suggest that in the case of family separation, maintaining regular contact, sharing a household with children at least part-time, or forming a new intact family may protect fathers from negative outcomes. Moreover, the combination of losing co-residential custody and having irregular contact with children seems to be especially harmful. Living together with some children, on the other hand, even if only part-time, might generally protect separated and blended-family fathers from negative outcomes after family separation, or even buffer the negative effect of loss of regular contact with other children. Yet, irrespective of their co-residency with biological children, blended-family fathers still show a worse outcome than bio fathers with stable families. In contrast to separated bio fathers, some of these blended-family fathers are living in an intact family again after separation, therefore having children living in their household once more. At the same time, however, they may still suffer from living apart from their children from previous relationships.

In previous cross-sectional, as well as longitudinal studies, active paternal involvement and regular contact with children was shown to be positively associated with well-being in fathers in general (15, 20, 47). However, family separation, and its concomitant changes in living arrangements, often leads to reduced father–child contact and relationship quality if children no longer live in the same household as their fathers (46, 47). Consequently, the emotional benefits of having children, including moment-to-moment positive emotions from childcare activities, fulfillment of fundamental psychological needs such as for meaning or affiliation, or sources of social relationships and support, could also diminish (24). Simultaneously, negative emotions, such as loneliness, feelings of loss, or missing one’s own children, might occur more often (24). Moreover, it might become more challenging to fulfill normative role expectations as an involved father. This might be accompanied by feelings of incompetence or failure, remorse, or role strain due to a perceived lack of control over decision making, dissatisfaction with visitation arrangements, or ambiguity about suitable behavior in the new role as a non-residential father (10, 24, 40, 48).

However, contrary to our hypothesis, reduced contact with children only mediated differences in well-being between bio fathers with stable families and some types of blended-family fathers, while other mechanisms seem to account for impairments in mental well-being in separated bio fathers. This is in line with previous longitudinal findings showing that diminished father–child relationship quality after family separation is not a mediator of higher distress in separated, non-resident fathers (46). Yet, in our sample, most separated bio fathers maintained regular contact with their children despite not living in the same household but were still markedly less involved in day-to-day childcare activities than fathers in stable families generally are. Thus, regular contact within the limits of visitation arrangements may not be sufficiently rewarding to compensate for the emotional costs of family separation, meaning that these fathers might still feel burdened by their lack of involvement in everyday childcare.

When interpreting these findings, the sociocultural background of our self-selected, mostly well-educated, healthy, Central European sample has to be taken into consideration. Due to the importance of contextual factors, culture-specific social norms, and father role identities for paternal involvement and well-being, caution should be exercised when generalizing these findings to other socioeconomic or cultural contexts. Particularly, evidence from non-Western, developing, poor, or non-democratic countries is still sparse, therefore not allowing a generalization of ours and other findings reported here to different sociocultural environments (24). Other limitations of the present study include the correlational nature of our data, which do not allow us to draw conclusions about the causality of our findings. Therefore, assumptions about the direction of associations have to be interpreted with caution before longitudinal data on the topic are available. We were not able to clearly rule out the possibility of reversed causation or selection: instead of fatherhood influencing paternal well-being, men with higher well-being might be more likely to stay together with their partner from the outset, more easily find a new partner to form a new intact family, gain child custody, or maintain regular contact with children after family separation. We tried to take into account some of the variety in “good provider potential” between fathers by including various sociodemographic and relationship aspects in our analyses.

Despite these limiting factors, our study provides considerable insights into the importance of contextual circumstances for a satisfying assumption of the father role. Specifically, our extensive characterization of different, complex forms of contemporary fatherhood results in a more detailed understanding of the challenges of different family constellations in which fatherhood is nowadays being lived out. The consideration of multiple facets of psychological well-being further allows us to make more general statements about the positive and negative impacts of these factors on overall paternal emotional well-being. Additionally, our large sample size not only enhances the generalizability of our findings but also allowed us to take into account an extensive number of potential confounding variables. This, in turn, helped us to clarify the reasons for differences between father types, while reducing the likelihood that unconsidered third variables were responsible for our findings.

As our findings indicate, fatherhood and the father–child relationship play a significant role in men’s emotional well-being, independently of other major social roles in a man’s life. The happiest fathers seem to be those who stay together with their family or maintain close relationships with their children after family separation. Thus, despite potential constraints resulting from active paternal involvement, a loss of this affiliative relationship seems to be more burdensome. Therefore, reasons for the reduction in father–child contact after family separation should be further determined. Beyond more static influences, such as the father’s personality or his identity with the father role before family separation, the inclusion of men in child custody and living arrangements can help to ensure continued paternal involvement, regular contact, and close affiliative relationships after family separation (46, 47). Non-residential fathers sometimes report that they perceive mothers to act as gatekeepers of the father–child relationship, controlling fathers’ access to their non-resident children, and at worst, erecting barriers to father–child contact (48). In line with this, a new partnership of the mother has been shown to be associated with a decrease in father–child contact, while this occurs to a far lesser extent when the father forms a new partnership (3, 60). Thus, whether non-residential fathers who wish to be actively involved face difficulty in maintaining regular contact with their children might be contingent on the mother’s perception about the usefulness of her former partner as a caregiver, or the presence of formal visitation agreements (3, 60).

While current legal practice in Central European countries generally envisages shared child custody between father and mother after family separation, division of childcare activities and living arrangements is still far from egalitarian. In most families, the mother still acts as the main caregiver, leaving the father with visitation arrangements after family separation (61, 62). Thus, family and labor policies and legal practice should further facilitate the provision of egalitarian childcare by fathers who are willing to be involved from the outset, making it easier for them to remain in an active father role after family break-up. Additionally, building up services for this high-risk population could support fathers in finding individual solutions to become more engaged. Finally, society, researchers, and politicians should stop underestimating the role of children for paternal well-being and the role of fathers for child development. After all, even if being an involved parent can be challenging: a close father–child-relationship can be beneficial for paternal well-being and the healthy development of his children.

## Author Contributions

PW was involved in the planning of the study, conducted the acquisition, analysis, and interpretation of the data, and prepared the first draft of the manuscript, including figures and tables. UE developed the study design, contributed to the interpretation of the data, and critically evaluated and revised the manuscript for important intellectual content. All authors approved the final version of the manuscript for submission.

## Conflict of Interest Statement

The authors declare that the research was conducted in the absence of any commercial or financial relationships that could be construed as a potential conflict of interest. The reviewer AG and handling Editor declared their shared affiliation, and the handling Editor states that the process nevertheless met the standards of a fair and objective review.
